# Revisiting Information Detection and Energy Harvesting: A Power Splitting-Based Approach

**DOI:** 10.3390/e22121341

**Published:** 2020-11-26

**Authors:** Jaehong Kim, Won-Yong Shin, Xin Kang, Han Lim Lee, Jingon Joung

**Affiliations:** 1 School of Electrical and Electronics Engineering, Chung-Ang University, Seoul 06974, Korea; kjhct9606@cau.ac.kr (J.K.); hanlimlee@cau.ac.kr (H.L.L.); 2 Department of Computational Science and Engineering, Yonsei University, Seoul 03722, Korea; wy.shin@yonsei.ac.kr; 3 Center for Intelligent Networking and Communications (CINC), University of Electronic Science and Technology of China (UESTC), Chengdu 611731, China; kangxin@uestc.edu.cn

**Keywords:** energy efficiency, energy harvesting, information decoding, power-splitting, optimal policy

## Abstract

Wireless sensors are becoming essential in machine-type communications and Internet of Things. As the key performance metrics, the spectral efficiency as well as the energy efficiency have been considered while determining the effectiveness of sensor networks. In this paper, we present several power-splitting solutions to maximize the average harvested energy under a rate constraint when both the information and power are transmitted through the same wireless channel to a sensor (i.e., a receiver). More specifically, we first designed the optimal dynamic power-splitting policy, which decides the optimal fractional power of the received signal used for energy harvesting at the receiver. As effective solutions, we proposed two types of single-threshold-based power-splitting policies, namely, Policies I and II, which decide to switch between energy harvesting and information decoding by comparing the received signal power with some given thresholds. Additionally, we performed asymptotic analysis for a large number of packets along with practical statistics-based policies. Consequently, we demonstrated the effectiveness of the proposed power-splitting solutions in terms of the rate–energy trade-off.

## 1. Introduction

Wireless sensors are becoming crucial in realizing machine-type communications and Internet of Things (IoT). Such wireless sensors are expected to be energy-efficient to ensure a sufficiently long lifetime of devices. Sensors can harvest the ambient energy and judiciously optimize the usage of the harvested energy subjected to energy causality constraints (see in [1,2,3,4] and the references therein); this can help in further improving the energy efficiency in devices without replacing the batteries. Subsequent to this significant achievement related to energy harvesting (EH) in wireless communication systems, more recent works studied the use of beamforming techniques for multiple-input multiple-output (MIMO) systems to supply the harvested energy. This type of energy beamforming can be simultaneously adopted with well-studied information beamforming for wireless communications because the same wireless channel can be exploited for both EH and information decoding (ID) [5]. However, the concurrent realization of EH and ID encounters a practical challenge. This is because any electrical signal used to detect whether a modulated signal represents a binary value of either zero or one for ID should have some (or all) of its current diverted from being used for EH. To solve this practical issue, various attempts have been made in the literature [6,7,8,9,10,11,12,13,14,15,16,17] for multiplexing the two signals for EH and ID over the time domain via time sharing, frequency domain via frequency division, or power domain via power-splitting.

Studies on simultaneous ID and EH have indeed received a great deal of attention. A trade-off between the information rate and energy, namely, a rate–energy (R–E) trade-off, was investigated in [5] for the case where a transmitter broadcasts the information and power simultaneously, and two receivers perform ID and EH. In [6], two practical receiver architectures for simultaneous ID and EH, referred to as separate and integrated receiver architectures, were designed, and the achievable R–E trade-off was studied. Additionally, the separate receiver architecture was applied to various wireless communication systems, such as ultra-wideband systems [8], cooperative relaying systems [9,10,11,12,13,14], heterogeneous cellular network systems [15], and single- and multi-antenna systems in point-to-point communications [16,17]. In [12,13], several EH schemes were suggested to support device-to-device transmission in cooperative non-orthogonal multiple access systems and examined the outage performance of each scheme. To enhance the performance of cooperative relay systems, multiple power beacons were used to serve devices far away from the base station [14]. Moreover, the EH technique was used in heterogeneous cellular networks where small-cell base station transfers an energy-bearing signal to users in downlink phases [15]. More specifically, the fundamental R–E trade-off was examined for multiple access and multi-hop channels in [9]. A greedy switching policy operating either on ID or EH [10] was presented for an amplify-and-forward relaying system. Dynamic time sharing (DTS) based on instantaneous channel gain and interference power, which enabled the receiver to perform ID or EH over different symbols, was performed in [16] by exploiting interference signals as the EH source. It should be noted that when the full channel state information (CSI) is available at the transmitter, power control and scheduling can be performed at the transmitter. However, the implementation of DTS involves practical challenges: (1) DTS requires accurate symbol-level synchronization and (2) it consumes the overhead in switching time. In another point of view, several dynamic power splitting (DPS) policies were discussed in [17,18,19]. In [17], an optimal DPS scheme, which has two different paths for ID and EH, was presented to determine the power ratio of the split paths using full CSI information. Because the power splitting scheme is not a time-based architecture, it can avoid some of the aforementioned practical issues encountered by DTS. Additionally, it is known that DPS usually outperforms the DTS scheme in terms of R–E trade-off [17]. Furthermore, another practical issue in simultaneous wireless information and power transfer (SWIPT)-based wireless sensors can be attributed to the low computational capability of devices. Several IoT devices, except high-end devices, have poor computational capabilities owing to limited resources [20]. Unfortunately, numerous decision processes are required to implement the optimal strategies in DTS or DPS using full CSI information. Such a burden on low-end IoT devices leads to time delay in the decision-making strategies, thereby directly causing performance degradation.

Many studies have attempted to adopt SWIPT in each antenna configuration. Investigations on the R–E trade-off in SWIPT were generally described in single-input single-output (SISO) systems [6,16]. Addition to the SISO systems, the R-E region with single-input multiple-output (SIMO) and MIMO systems was also studied in [21,22]. The R-E region considering multi-user interference was analyzed in [23]. Considering the aspects of a multi-user interference channel, the authors of [24] studied the case where a SIMO system was applied. Similar work on multiple-input single-output (MISO) systems was suggested in [25]. Several studies proposed SWIPT transceiver design for multi-user interference scenarios in MIMO systems [26,27,28]. For the secrecy problems, the authors of [29,30] evaluated the secrecy rates in SIMO, and the works in [31,32] also examined it in MISO systems. Other advanced works examined the beamforming techniques in several MISO [33,34,35] and MIMO systems [36,37,38].

In this paper, we present several power-splitting solutions for a sensor network scenario where the information and power are transmitted through the same wireless channel to a sensor (i.e., a receiver end). Accordingly, we first formulated a constrained optimization problem to maximize the average harvested energy under a minimum average rate constraint. (In a preliminary version [39] of this work, we defined the same problem and proposed its solutions. This study subsumes the work in [39] while making new non-trivial contributions analytically and numerically.) To optimally solve the problem, we designed the optimal DPS policy, which decides the optimal *fractional power* of the received signal used for EH and the remaining fraction used for ID under the assumption that full CSI is available. Our DPS solution indicates that if the target rate *R* increases, the receiver makes suitable adjustment by increasing the fractional power used for ID, implying that the remaining fraction used for EH is decreased accordingly. In addition, the practical challenge encountered by the optimal DPS solution in terms of realization motivates us to present simple threshold-based power-splitting (TPS) policies for each implementation. Specifically, under the same objective function as that of the DPS case, we restricted the DPS variables to be binary, i.e., they takes two options either ID or EH. We then determined an asymptotically optimal solution to the TPS problem for a large number of packets when only the causal CSI is available by proposing two classes of *single-threshold* TPS policies, namely, Policies I and II. Furthermore, to alleviate the impractical channel conditions, we introduced statistics-based policies to ensure that our EH–ID receiver operates based on the *statistics* of channel gains without instantaneous CSI. Through numerical evaluation, we demonstrated the effectiveness of our power-splitting policies by empirically characterizing a fundamental trade-off between the information rate and harvested energy. Our numerical findings elucidate that (i) the optimal DPS policy is always dominant in terms of the R–E trade-off for all simulation settings, such as the distribution types of the channel gain, and (ii) the performance of the TPS policy with the optimal threshold is consistently superior to that of another TPS policy with the threshold designed according to the channel gain statistics. Our methodology explains how an EH–ID receiver can be effectively designed for ease of implementation while guaranteeing the (asymptotic) optimality of the performance.

The remainder of this paper is organized as follows. In Section 2, the system and signal models are described. The optimal DPS solution and several TPS policies are presented in Section 3 and Section 4, respectively. Numerical results are discussed in Section 5. Finally, we summarize the paper with concluding remarks in Section 6.

## 2. System and Signal Models

**Figure 1:**
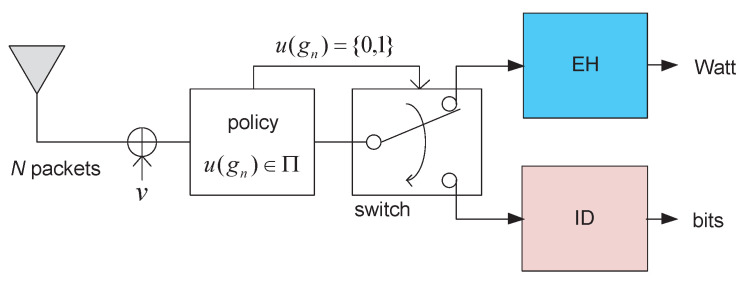
Energy harvesting (EH) and information decoding (ID) receiver structure with a threshold-based controller and packet-by-packet switch.

We consider a slotted wireless communication system consisting of a single-antenna transmitter (e.g., an access point), which transmits both the data and energy, and a single-antenna receiver (e.g., a sensor) for EH and ID, as shown in Figure 1. Transmissions take place over *N* slots, where, in each time slot, a packet of *L* symbols is transmitted. The baseband received signal for the *l*th transmit symbol in the *n*th packet is given by

(1) $$y\left[n,l\right]=h\left[n\right]\sqrt{P}x\left[n,l\right]+v\left[n,l\right]$$

for l=1,⋯,L and n=1,⋯,N. Here, h[n] is a complex-valued channel coefficient for the *n*th packet that remains invariant in each packet but can change over packets; P>0 is the fixed average transmit power over all packets; v[n,l] is an additive white Gaussian noise with zero mean and variance, i.e., $v\left[n,l\right]∼\mathrm{CN}\left(0,\sigma_{v}^{2}\right),\forall n$; x[n,l] is the transmitted symbol that is independent over *n* and *l*, and conforms to the Gaussian distribution to maximize the mutual information, i.e., x[n,l]∼CN(0,1),∀n, and ∀l.

We denote the received signal power for each symbol in the *n*th packet by the energy detector as

(2) $$g_{n}=P{\left|h[n]\right|}^{2}.$$

If the receiver performs ID with the full knowledge of the channels, then it can achieve the information rate expressed as the mutual information [40]

(3) $$I\left(g_{n}\right)=\log\left(1+\frac{g_{n}}{\sigma_{v}^{2}}\right)\;b/s/\mathrm{Hz},$$

where the logarithm takes the base of two, unless otherwise specified. If the receiver performs EH, it can obtain the power given by [6]

(4) $$e_{n}=\eta g_{n}\;J/s,$$

where 0<η≤1 is the energy conversion efficiency when converting the wireless power to the harvested energy that is stored in energy storage devices such as a battery or supercapacitor. Typically, it follows that 0<η≪1 owing to the dissipation of energy in the form of heat. The receiver performs either ID or EH based on the policy $u(g_{n})$, which will be rigorously discussed throughout this study.

## 3. Optimal DPS Solution

In this section, we introduce the optimal solution to the problem of maximizing the average harvested energy, *E*, subject to a minimum average achievable rate, *R*, for reliably decoding the data. Accordingly, we first assume that *full CSI* is available, i.e., the received signal power, $g_{n},n=1,\cdots ,N$, is available as input in the optimization problem, where *n* denotes the packet index; this is also referred to as the offline approach. We shall later assume that *only causal CSI* is available; this is referred to as the online approach.

First, we tackle a general DPS problem. In DPS, the variables to be optimized are the power splitting variables $0\leq s_{n}\leq 1$, ∀n∈{1,⋯,N}. The variable $s_{n}$ denotes the fractional power of the received signal used for EH, while the remaining $\left(1-s_{n}\right)$ fraction is used for ID. Thus, under the full CSI assumption, our *DPS problem* can be formulated as

(5) $$\begin{array}{ccc} & \max_{\left\{0\leq s_{n}\leq 1\right\}} & E≜\frac{1}{N}\sum_{n=1}^{N}\eta g_{n}s_{n} \end{array}$$

(6) $$\begin{array}{ccc} & s.t. & \frac{1}{N}\sum_{n=1}^{N}I\left(g_{n}\left(1-s_{n}\right)\right)\geq R. \end{array}$$

where *R* is the target rate. Throughout this study, we assumed that a rateless code [41] was employed in each packet and the receiver could accumulate the mutual information by performing joint decoding over the received packets. We denote the optimal DPS solution as $\left\{s_{n}^{*}\right\}$ and the maximum average harvested energy as $E_{\mathrm{DPS}}^{*}$. The optimal DPS solution is essentially derived from [17] and is stated in the following theorem.

**Theorem** **1.** *Suppose that full CSI is available. Then, for R>0, the solution to the DPS problem in (5) and (6) is given by*

(7) $$\begin{array}{c} s_{n}^{*}=\left\{\begin{array}{cc} 1-\frac{\tau }{g_{n}}, & \mathrm{if}\;g_{n}\geq \tau \\ 0, & \mathrm{if}\;g_{n}<\tau , \end{array}\right. \end{array}$$

*where $\tau ≜\frac{\lambda }{\eta \ln2}-\sigma_{v}^{2}>0$ and λ is a Lagrangian multiplier that satisfies $\lambda >\sigma_{v}^{2}\eta \ln2$.*

**Proof.**  The DPS problem is a convex optimization problem. By solving the dual problem along with the Karush–Kuhn–Tucker conditions, (7) can be obtained. The detailed proof is provided in Appendix A for completeness. □

Based on Theorem 1, the following insightful observations were made according to the received signal power, $g_{n}$.

**Remark** **1.** The constant τ acts as a threshold that determines the characteristics of the DPS solution. More specifically, the receiver performs ID (i.e., $s_{n}^{*}=0$) if the channel gain is too small, i.e., if $g_{n}<\tau$. In contrast, the receiver performs a non-trivial DPS for EH and ID (i.e., $0<s_{n}^{*}<1$), i.e., $g_{n}\geq \tau$, owing to $\lambda >\sigma_{v}^{2}\eta \ln2$. Thus, the optimal DPS solution never harvests all energy in any one slot irrespective of the value of $g_{n}$.

The solution in (7) can be interpreted as follows. If the target rate, *R*, increases, then $s_{n}^{*}$ needs to decrease for some *n* to satisfy (6); in turn, λ increases, and consequently, $s_{n}^{*}$ in (7) decreases for all *n*. In other words, if *R* increases, then the receiver makes adjustments by increasing the fractional power used for ID and decreasing the remaining fraction used for EH. The power ratio for ID decreases if the channel gain, $g_{n}$, is high because the high channel gain is sufficient for achieving the target rate *R* with a small amount of the received power; meanwhile, the power ratio for EH increases as the remaining signal is used for EH. When there are no feasible solutions for $\left\{s_{n}\right\}$, i.e., $\frac{1}{N}\sum_{n=1}^{N}I\left(g_{n}\right)<R$, ID is not performed at the receiver.

By observing (7) and (6), we establish the following corollary.

**Corollary** **1.** If R=0, then the receiver only performs EH, i.e., $s_{n}^{*}=1,\forall n$. In contrast, if R>0, then the receiver performs a mixture of EH and ID. Especially, the nth packet with its channel gain, $g_{n}<\frac{\lambda -1}{\eta \ln2}≜\tau$, is used only for ID, while the $n^{\prime }$th packet with its channel gain, $g_{n^{\prime }}\geq \tau$, is used for both EH and ID.

**Proof.**  Refer to Appendix B for the proof. □

Moreover, we discuss some practical situations related to the optimal power splitting as follows.

**Figure 2:**
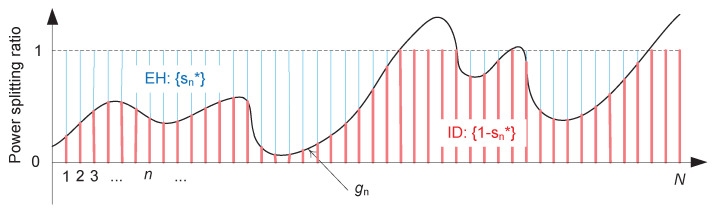
Illustration of optimal power splitting ratios, $s_{n}^{*}$ and $\left(1-s_{n}^{*}\right)$, for EH and ID, respectively, over *N* packets.

**Remark** **2.** *As illustrated in Figure 2 as a motivating example, the optimal power splitting in (7) involves the following three practical issues:*

- *All N packets and their channels should be stored in a controller/buffer to determine the power splitting ratio $\left\{s_{n}\right\}$. Note that τ is a function of λ, which is a function of all $g_{n}$.*
- *Power splitting for each packet requires a high-speed and highly accurate power splitter.*
- *Computing λ may cause a significant delay and requires high computational complexity.*

Although the optimal power splitting in (7) has a practical challenge in terms of realization, it informs us of providing a performance bound, and furthermore allows us to obtain insights on the structure of the policy based on a threshold. In the next section, we present simple TPS policies for ease of implementation.

## 4. Optimal TPS Solutions

In this section, to ensure that EH can be easily implemented in practice, we present several TPS policies. We also present asymptotically optimal TPS solutions for a large number of packets, *N*.

### 4.1. Problem Formulation

We propose an adaptive form of power splitting. To further simplify the implementation, we focus on power switching that only depends on the current channel gain instead of the current packet index. This allows the TPS solution to be stored as a policy with a smaller storage size.

Specifically, in our TPS problem, the objective function and constraint are essentially the same as those in the DPS problem, but we restrict the DPS variables to be binary that accept the values of 0 (for ID only) or 1 (for EH only). To distinguish from the real-valued DPS variables $\left\{s_{n}\right\}$, we refer to the new binary variables as TPS variables, denoted as $\left\{u_{n}\right\}$. To reduce the storage of the solution, leading to easier implementation, we design the TPS variables, $u_{n}$, such that they only depend on the channel gain, i.e., $u_{n}=u\left(g_{n}\right)$, where u(·)∈Π is a function or *policy* and Π is the feasible policy space consisting of all possible functions that take a positive value as input and return a binary value as output. Then, the TPS problem aims to find the optimal policy u(g): (To simplify the notations, $g_{n}$ will be written as *g* if dropping *n* does not cause any confusion.)

(8) $$\begin{array}{ccc} & \max_{u(g)\in \Pi } & E≜\frac{1}{N}\sum_{n=1}^{N}\eta g_{n}u\left(g_{n}\right) \end{array}$$

(9) $$\begin{array}{ccc} & s.t. & \frac{1}{N}\sum_{n=1}^{N}I\left(g_{n}\left(1-u\left(g_{n}\right)\right)\right)\geq R. \end{array}$$

We denote the optimal TPS policy as $u^{*}\left(g\right)$ and the maximum average harvested energy as $E_{\mathrm{TPS}}^{*}$. Note that $E_{\mathrm{TPS}}^{*}\leq E_{\mathrm{DPS}}^{*}$ because any feasible TPS solution is a feasible DPS solution but the converse is not necessarily true.

Before investigating the optimal policy for the TPS problem, we provide an intuitive discussion on the possible relationship between the optimal DPS and TPS solutions. From Remark 1, $s_{n}^{*}$ does not take the value of 1. Thus, it is not immediately clear how the optimal DPS solution, $\left\{s_{n}^{*}\right\}$, translates to the optimal TPS policy, $u^{*}\left(g\right)$. A reasonable conjecture can be derived considering that $u^{*}\left(g\right)=0$ for small *g* to be consistent with (7). However, this conjecture is not true in general. In other words, it is possible that $u^{*}\left(g\right)=0$ for some large *g*, while $u^{*}\left(g\right)=1$ for some small *g*. For the DPS solution, there exists a single threshold for *g* that determines whether $u^{*}\left(g\right)=0$ or $u^{*}\left(g\right)=1$. It is, however, intuitively unclear whether there may exist a single or multiple thresholds for the TPS policy.

### 4.2. Class of TPS Policies

We note that the TPS problem is an integer program that, in general, is difficult to solve exactly. In this section, we aim to find an asymptotically optimal solution for the TPS problem for large *N* under any given channel condition. We shall see that the optimal solution is well structured and provides insights on solving the offline problem where only the causal CSI is available.

Without loss of generality, by taking the value of 1 in set S and 0 in set T, we can express the TPS policy as follows.

(10) $$u\left(g_{n}\right)=\left\{\begin{array}{cc} 1\;\left(\mathrm{EH}\right), & \mathrm{if}\;g_{n}\in S\\ 0\;\left(\mathrm{ID}\right), & \mathrm{if}\;g_{n}\in T. \end{array}\right.$$

**Figure 3:**
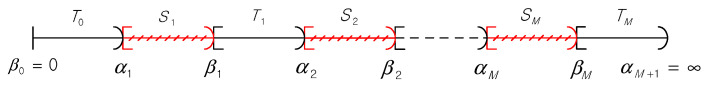
Illustration of two sets $S={⋃}_{m=1}^{M}S_{m}$ and $T={⋃}_{m=0}^{M}T_{m}$ for the TPS policy with *M*-pair thresholds.

Note that sets S and T are possibly non-contiguous. This implies that the TPS policy has *M*-pair thresholds if S and T can be expressed as at least *M* and M+1 contiguous regions, i.e., $S={⋃}_{m=1}^{M}S_{m}$ and $T={⋃}_{m=0}^{M}T_{m}$, where $S_{m}$ and $T_{m}$ are the contiguous sets for all *m*. Specifically, as depicted in Figure 3, we have

(11) $$\begin{array}{ccc} S_{m} & ≜ & \left\{g:\alpha_{m}\leq g<\beta_{m}\right\} \end{array}$$

(12) $$\begin{array}{ccc} T_{m} & ≜ & \left\{g:\beta_{m}\leq g<\alpha_{m+1}\right\} \end{array}$$

such that $\beta_{0}\leq \alpha_{1}<\beta_{1}<\alpha_{2}<\beta_{2}<\cdots <\beta_{M}\leq \alpha_{M+1}$, with $\beta_{0}=0$ and $\alpha_{M+1}=\infty$. Thus, the policy u(g)∈Π can be uniquely determined by the *M*-pair variables $\left\{\alpha_{m},\beta_{m}\right\}$ for m=1,…,M. Our policy is said to have a *single threshold* if M=1 and $\alpha_{1}=0$ or $\beta_{1}=\infty$.

### 4.3. Two Single-Threshold Policies

**Figure 4:**
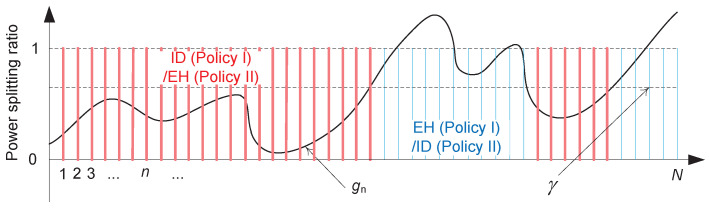
Illustration of threshold-based power-splitting (TPS) policies I and II.

Before stating our new analytical finding, we characterized two important classes of single-threshold TPS policies as follows,

(13) $$\begin{array}{ccc} & \mathrm{Policy}\;I:\; & u_{1}\left(g_{n}\right)=\left\{\begin{array}{cc} 1\;\left(\mathrm{EH}\right), & \mathrm{if}\;g_{n}\geq \gamma_{1}\\ 0\;\left(\mathrm{ID}\right), & \mathrm{if}\;g_{n}<\gamma_{1} \end{array}\right. \end{array}$$

(14) $$\begin{array}{ccc} & \mathrm{Policy}\;\mathrm{II}:\; & u_{2}\left(g_{n}\right)=\left\{\begin{array}{cc} 0\;\left(\mathrm{ID}\right), & \mathrm{if}\;g_{n}>\gamma_{2}\\ 1\;\left(\mathrm{EH}\right), & \mathrm{if}\;g_{n}\leq \gamma_{2}, \end{array}\right. \end{array}$$

which are illustrated in Figure 4.

It should be noted that Policies I and II are realized with $\left\{\alpha_{1},\beta_{1}\right\}=\left\{\gamma_{1},\infty \right\}$ and $\left\{\alpha_{1},\beta_{1}\right\}=\left\{0,\gamma_{2}\right\}$, respectively. Let us denote the maximum harvested energy achieved in the TPS problem, where we restrict to Policies I and II, as $E_{1}$ and $E_{2}$, respectively. Then, for each policy, there exists only one parameter given by threshold $\gamma_{i}$ for i∈{1,2}. Therefore, $E_{i}$ can be easily obtained by setting the constraint (9) to the equality and solving for $\gamma_{i}$ accordingly because $E_{i}$ can be further increased otherwise.

### 4.4. Asymptotic Analysis

In this subsection, we present the *asymptotic* analysis built upon the analytical findings of our previous work [39]. More specifically, we show that the optimality of Policy I is valid for a large number of packets, *N*. We proceed with providing a sufficient condition for the asymptotic optimality of Policy I as follows.

**Theorem** **2.** For any channel gain, $\left\{g_{n}\right\}$, Policy I solves the TPS problem within the energy gap $\epsilon^{\prime }\left(N\right)\geq 0$ of the optimal policy, i.e., $E_{1}\geq E_{\mathrm{TPS}}^{*}-\epsilon^{\prime }\left(N\right)$, along with the asymptotic property stating that for large N, $\epsilon^{\prime }\left(N\right)=O\left(1/N\right)$.

**Proof.**  See Appendix C for the proof. □

From Theorem 2, it asymptotically follows that $\epsilon^{\prime }\left(N\right)=O\left(1/N\right)\to 0$ for large *N*. Thus, Policy I is asymptotically optimal over the class of any policy as *N* increases.

### 4.5. Statistics-Based Policies

Although Policy I was found to be optimum, we still need to determine the optimal threshold, $\gamma_{1}^{*}$, for Policy I. The receiver should be aware of all $g_{n}$ to find the minimum $\gamma_{1}$ that satisfies constraint (9). In practice, however, the proposed EH–ID receiver in Figure 1 operates packet-by-packet; therefore, it is difficult for the receiver to know all $g_{n}$ before data detection. To resolve this issue, we introduce a way of determining $\gamma_{1}^{*}$ based on the *statistics* of channel gains.

Under the *wide-sense stationary* assumption, we rewrite (8) and (9) with a single threshold as follows,

(15) $$\begin{array}{cc} \max_{\gamma_{i}\geq 0}\;\; & \eta \;E\left[gu_{i}\left(g\right)\right]\\ s.t.\;\;\; & E\left[I\left(g(1-u_{i})\right)\right]\geq R, \end{array}$$

where i∈{1,2}, and E[·] represents the expectation over *g*. Equation (15) can further be rewritten as

(16) $$\begin{array}{ccc} & \max_{\gamma_{1}\geq 0} & \;\eta \int_{\gamma_{1}}^{\infty }gf\left(g\right)dg \end{array}$$

(17) $$\begin{array}{ccc} & s.t.\; & \int_{0}^{\gamma_{1}}\log_{2}\left(1+g\right)f\left(g\right)dg\geq R \end{array}$$

and

(18) $$\begin{array}{ccc} & \max_{\gamma_{2}\geq 0} & \;\eta \int_{0}^{\gamma_{2}}gf\left(g\right)dg \end{array}$$

(19) $$\begin{array}{ccc} & s.t.\; & \int_{\gamma_{2}}^{\infty }\log_{2}\left(1+g\right)f\left(g\right)dg\geq R \end{array}$$

for policies I and II, respectively, where f(g) is the probability density function (PDF) of *g*, and without loss of generality, $\sigma_{v}^{2}=1$. As $\gamma_{1}$ increases in (16) and (17), the objective function in (16) decreases monotonically, while the left-hand side of constraint (17) increases monotonically. On the other hand, as $\gamma_{2}$ increases in (18) and (19), the objective function in (18) increases monotonically, while the left-hand side of constraint (19) decreases monotonically. Therefore, the optimal $\gamma_{1}^{*}$ and $\gamma_{2}^{*}$ are designed by solving the equalities in the constraints as follows,

(20) $$\begin{array}{cc} \int_{0}^{\gamma_{1}^{*}}\log_{2}\left(1\;+\;g\right)f\left(g\right)dg=R, & \mathrm{for}\;\gamma_{1}^{*}\;\mathrm{of}\;\mathrm{Policy}\;I,\\ \int_{\gamma_{2}^{*}}^{\infty }\log_{2}\left(1\;+\;g\right)f\left(g\right)dg=R, & \mathrm{for}\;\gamma_{2}^{*}\;\mathrm{of}\;\mathrm{Policy}\;\mathrm{II}. \end{array}$$

Evidently, it follows that $\gamma_{1}^{*}=0$ and $\gamma_{2}^{*}=\infty$ if R=0; however, it is difficult to derive a closed form expression of the optimal threshold for general f(g) with R>0. Nevertheless, the optimal threshold can always be determined from (20), at least numerically.

In general, it is difficult to analytically find the optimal threshold, except for a few special cases of f(g). For example, we consider a monomial function of f(g) as

(21) $$f\left(g\right)=\left\{\begin{array}{cc} Kg^{\alpha }, & K_{1}\leq g\leq K_{2}\\ 0, & \mathrm{otherwise}, \end{array}\right.$$

where α is an integer, $K=\frac{\alpha +1}{K_{2}^{\alpha +1}-K_{1}^{\alpha +1}}$ if α≠−1, and $K=\frac{1}{\ln K_{2}-\ln K_{1}}$ if α=−1 so that f(g) becomes a PDF. Note that $K_{1}>0$ if α<0. As a special case where α=0, we obtain specific solutions from (20) as follows,

(22) $$\begin{array}{ccc} \gamma_{1}^{*} & = & \exp\left(W_{0}\left(\frac{\xi_{1}}{\exp(1)}\right)+1\right)-1 \end{array}$$

(23) $$\begin{array}{ccc} \gamma_{2}^{*} & = & \left\{\begin{array}{cc} \exp\left(W_{0}\left(\frac{\xi_{2}}{\exp(1)}\right)+1\right)-1, & \mathrm{if}\;\xi_{2}\geq -1\\ 0, & \mathrm{otherwise}, \end{array}\right. \end{array}$$

where $W_{0}$ is a Lambert W function, $\xi_{1}=R\left(K_{2}-K_{1}\right)\ln2+\left(K_{1}+1\right)\ln\left(K_{1}+1\right)-K_{1}-1$, and $\xi_{2}=-R\left(K_{2}-K_{1}\right)\ln2+\left(K_{2}+1\right)\ln\left(K_{2}+1\right)-K_{2}-1$.

However, the optimal policy is still unclear. To find an optimal policy for the statistical case, we compare $E_{1}$ and $E_{2}$ by deriving them using the following equations,

(24) $$\begin{array}{ccc} E_{1}^{*} & = & \eta \;\int_{{\gamma_{1}}^{*}}^{\infty }gf\left(g\right)\;dg \end{array}$$

(25) $$\begin{array}{ccc} E_{2}^{*} & = & \eta \;\int_{0}^{\gamma_{2}^{*}}gf\left(g\right)\;dg. \end{array}$$

It is difficult to find a necessary and sufficient condition for either $E_{1}\geq E_{2}$ or $E_{2}>E_{1}$ with a general PDF of the received signal power, i.e., f(g). In our study, we establish the following theorem for the monomial function f(g).

**Theorem** **3.** *Policy I is optimal if the PDF of the received signal power is a monomial function in (21).*

**Proof.**  See Appendix D for the proof. □

## 5. Numerical Evaluation and Discussion

In this section, we perform numerical evaluation via intensive simulations to empirically validate the effectiveness of our TPS policies by applying the monomial function in (21) and exponential function as statistics of the channel gains.

### 5.1. Simulation Environment

We first describe the simulation environment as follows. We evaluate the performance of EH and ID for different power splitting policies by varying the target rate R≥0. Accordingly, we numerically characterize an R–E trade-off for each policy. The power conversion efficiency was set to η=0.6. Each point on the EH–ID trade-off was numerically obtained by transmitting $N=10^{6}$ packets. We considered Rayleigh fading channels, i.e., $h\left[n\right]∼\mathrm{CN}\left(0,\sigma_{h}^{2}\right)$ for the *n*th packet to model a typical channel in wireless communications. The PDF, f(g), of $g={P|h\left[n\right]|}^{2}$ can be then given by

(26) $$f\left(g\right)=\left\{\begin{array}{cc} \frac{1}{P\sigma_{h}^{2}}\exp\left(-\frac{g}{P\sigma_{h}^{2}}\right), & \mathrm{if}\;g\geq 0,\\ 0, & \mathrm{otherwise}. \end{array}\right.$$

### 5.2. Numerical Results

**Figure 5:**
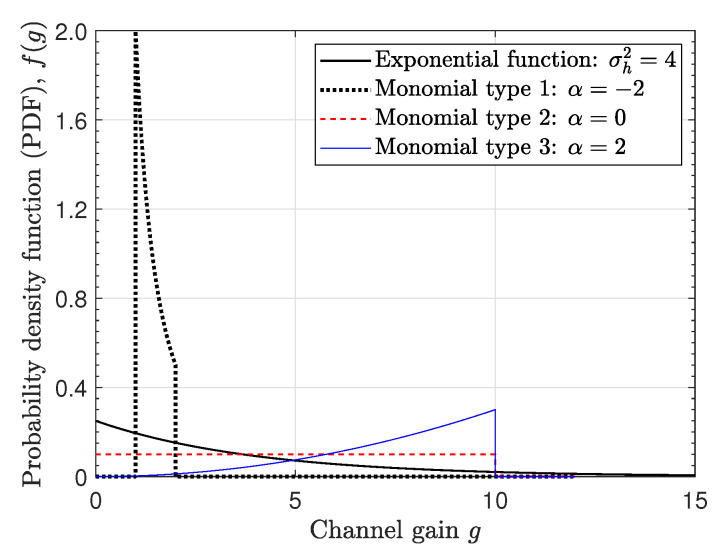
Probability density functions (PDFs) of channel gain *g* according to four types of channels.

In our simulations, we considered four types of PDFs of channel gain *g* as follows (see Figure 5).

(1) Exponential function in (26): Rayleigh fading with $\sigma_{h}^{2}=4$.
(2) Monomial function in (21): type-1 with low gain dominant channels for α=−2, $K_{1}=1$, and $K_{2}=2$.
(3) Monomial function in (21): type-2 with uniform gain channels for α=0, $K_{1}=0$, and $K_{2}=10$.
(4) Monomial function in (21): type-3 with high gain dominant channels for α=2, $K_{1}=0$, and $K_{2}=10$.

**Figure 6:**
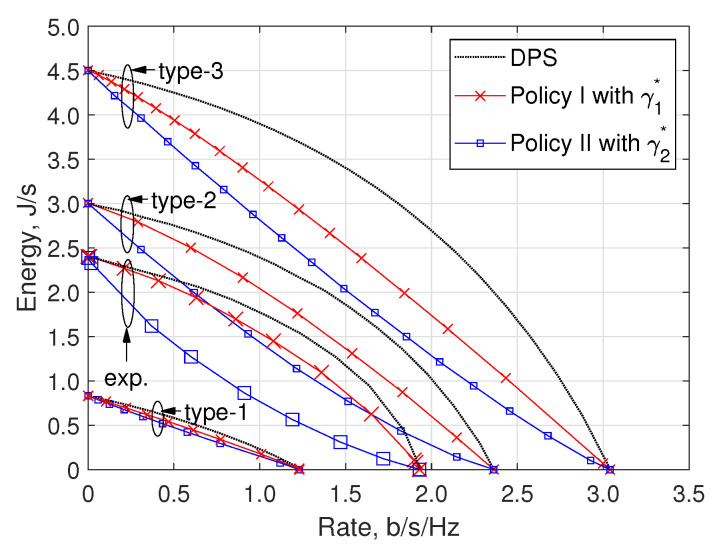
R–E trade-off according to four types of PDFs of channel gain, where three power splitting policies are adopted for each type.

Now, we evaluate the performance of the following three power-splitting policies: (i) the optimal DPS policy using $s_{n}^{*}$ in (7); (ii) the TPS policy with the optimal threshold $\gamma_{1}^{*}$ in (22) (i.e., Policy I with $\gamma_{1}^{*}$); and (iii) another TPS policy with the optimal threshold $\gamma_{2}^{*}$ in (23) (i.e., Policy II with $\gamma_{2}^{*}$). Figure 6 illustrates the R–E trade-off region according to the aforementioned four types of PDFs of channel gain, where three power splitting policies are adopted for each type. The results clearly exhibit that the optimal DPS policy achieves the highest harvested energy for a given information rate, i.e., the outermost boundary of the trade-off region, irrespective of the PDF type. In other words, the optimal DPS policy reveals the best R–E trade-off. It can be observed that the performance of Policy I with $\gamma_{1}^{*}$ is consistently superior to that of Policy II with $\gamma_{2}^{*}$; this can be attributed the fact that Policy II with $\gamma_{2}^{*}$ is naïvely designed based on the statistics of channel gains when no CSI is available. Moreover, it can be observed that the performance on the R–E trade-off improves when α increases (i.e., the case of high gain dominant channels).

## 6. Concluding Remarks

In this paper, we presented several power-splitting policies by formulating the harvested energy maximization problem under a minimum rate constraint for wireless point-to-point communication systems where both the information and power are transmitted through the same wireless channel to a sensor. To optimally solve the problem, we first designed an optimal DPS policy wherein the receiver makes adjustments by increasing the fractional power for ID and decreasing the remaining fraction for EH when the target rate increases. In addition, as effective solutions, we proposed two types of single-threshold TPS policies, policies I and II. Subsequently, we presented an asymptotic analysis based on our previous analytical findings, which proves that the optimality of policy I is valid when the number of packets is sufficiently large. Furthermore, we introduced statistics-based policies to ensure that the EH–ID receiver operates according to channel gain statistics without instantaneous CSI. The numerical results demonstrated that the optimal DPS policy always exhibits the best performance in terms of the R–E trade-off. It was also empirically shown that policy I with the optimal threshold consistently outperforms policy II with another threshold determined by the channel gain statistics in various simulation settings.

## Abbreviations

The following abbreviations are used in this manuscript.

CSI	channel state information
DPS	dynamic power splitting
DTS	dynamic time sharing
EH	energy harvesting
ID	information decoding
MIMO	multiple-input multiple-output
MISO	multiple-input single-output
PDF	probability density function
R–E	rate–energy
SIMO	single-input multiple-output
SISO	single-input single-output
SWIPT	simultaneous wireless information and power transfer
TPS	threshold-based power-splitting

## Appendix A. Proof of Theorem 1

**Proof.** The optimization problem in (5) and (6) is formulated as a Lagrangian problem without any constraint; thus, it can be expressed as

(A1) $$\max_{\left\{0\leq s_{n}\leq 1,\lambda \geq 0\right\}}\frac{1}{N}\sum_{n=1}^{N}\eta g_{n}s_{n}+\lambda \left(\frac{1}{N}\sum_{n=1}^{N}I\left(g_{n}\left(1-s_{n}\right)\right)-R\right).$$

Because (A1) is a concave optimization problem without any constraint, we can find the optimal $s_{n}^{*}$ from the solution that enables the first derivative of the objective function with respect to $s_{n}$ to be zero as follows (Note that the result in (A2) is identical to that in [17], wherein the solution $s_{n}^{*}$ is obtained by maximizing ID with the EH constraint.),

(A2) $$s_{n}^{*}={\left[1-\frac{1}{g_{n}}\left(\frac{\lambda }{\eta \ln2}-\sigma_{v}^{2}\right)\right]}_{0}^{1},\;\forall n\in \left\{1,\ldots ,N\right\},$$

where ${\left[x\right]}_{a}^{b}$ takes *x* if it lies between *a* and *b*; otherwise, it takes the closest boundary *a* or *b*. The Lagrangian multiplier, λ, is derived from (6). From (6), if R>0, then at least one $s_{n}^{*}$ should be greater than zero to satisfy (6). Therefore, from (A2), it can be demonstrated that $\tau =\frac{\lambda }{\eta \ln2}-\sigma_{v}^{2}$ should be positive, and $s_{n}^{*}=0$ if $g_{n}<\tau$ and $0<s_{n}^{*}<1$ if $g_{n}\geq \tau$. This completes the proof of this theorem. □

## Appendix B. Proof of Corollary 1

**Proof.** From (7), $s_{n}^{*}=1,\forall n$, by setting R=0. In contrast, if R>0, then at least one $s_{n}^{*}$ should be greater than zero to satisfy (6). Thus, from (A2), it can be demonstrated that $s_{n}^{*}=0$ if $g_{n}<\frac{\lambda -1}{\eta \ln2}$ and $0<s_{n}^{*}<1$ if $g_{n}\geq \frac{\lambda -1}{\eta \ln2}$. This completes the proof of this corollary. □

## Appendix C. Proof of Theorem 2

**Proof.** Consider the TPS policy with a single threshold. From Theorem 1 in [39], it can be demonstrated that $E_{1}\geq E_{2}-\left|\epsilon \left(N\right)\right|$, where $E_{1}$ and $E_{2}$ are the maximum energy of policies I and II, respectively, which are harvested from *N* packets in S∪T. Following the procedure in the proof of Lemma 1 in [39], it can be observed that the loss of harvested energy due to switching the optimal TPS policy to policy I is not greater than |ϵ(N)|. Therefore, if we implement policy I, we can readily derive the maximum gap of harvested energy between the global optimal TPS and global policy I as $\epsilon^{\prime }\left(N\right)=\left|\epsilon \left(N\right)\right|\geq 0$; thus, we have

$$E_{1}\geq E_{TPS}^{*}-\epsilon^{\prime }\left(N\right).$$

From Theorem 1 in [39], ϵ(N)→0 as N→∞. Assuming N→∞, we can further assume that the number of packets with channel gain in S∪T increases without a limit, i.e., N→∞. Therefore, we finally have $\epsilon^{\prime }\left(N\right)\to 0$, which completes the proof of this theorem. □

## Appendix D. Proof of Theorem 3

We basically use the convex function calculus (CFC) to prove this theorem.

*CFC: If f(x) is convex (i.e., $f^{\prime \prime }\left(x\right)>0$) and h(x) is convex and non-decreasing (i.e., $h^{\prime }\left(x\right)>0$ and $h^{\prime \prime }\left(x\right)>0$), then h(f(x)) is convex. However, if f(x) is concave (i.e., $f^{\prime \prime }\left(x\right)<0$) and h(x) is convex and non-increasing (i.e., $h^{\prime }\left(x\right)\leq 0$ and $h^{\prime \prime }\left(x\right)>0$), then h(f(x)) is convex.*

**Proof.** For simplicity, without loss of generality, we assume the perfect conversion efficiency of power, i.e., η=1. Using PDF f(g) in (21), we can derive $E_{1}$ and $E_{2}$ as follows,

$$\begin{array}{ccc} E_{1} & = & \int_{\gamma_{1}}^{K_{2}}Kgg^{\alpha }dg\\ & = & \left\{\begin{array}{cc} \frac{K}{\alpha +2}\left(K_{2}^{\alpha +2}-\gamma_{1}^{\alpha +2}\right) & \mathrm{if}\;\alpha \neq -2,\\ K\left(\ln K_{2}-\ln\gamma_{1}\right) & \mathrm{if}\;\alpha =-2, \end{array}\right. \end{array}$$

$$\begin{array}{ccc} E_{2} & = & \int_{K_{1}}^{\gamma_{2}}Kgg^{\alpha }dg\\ & = & \left\{\begin{array}{cc} \frac{K}{\alpha +2}\left(-K_{1}^{\alpha +2}+\gamma_{2}^{\alpha +2}\right) & \mathrm{if}\;\alpha \neq -2,\\ K\left(-\ln K_{1}+\ln\gamma_{2}\right) & \mathrm{if}\;\alpha =-2, \end{array}\right. \end{array}$$

where $\gamma_{1}$ and $\gamma_{2}$ are the functions of *R* satisfying

(A3) $$\begin{array}{ccc} \gamma_{1} & \;s.t.\; & R=\int_{K_{1}}^{\gamma_{1}}\log_{2}\left(1+g\right)Kg^{\alpha }dg, \end{array}$$

(A4) $$\begin{array}{ccc} \gamma_{2} & \;s.t.\; & R=\int_{\gamma_{2}}^{K_{2}}\log_{2}\left(1+g\right)Kg^{\alpha }dg, \end{array}$$

respectively. For the integration in (A4), using the integration formulation below

(A5) $$\begin{array}{ccc} \int \log_{2}\;\;\;\;\;\;\;\;\; & & \left(1\;+\;x\right)x^{\alpha }dx\\ & \;=\; & \left\{\begin{array}{cc} \frac{1}{\ln2}\frac{1}{1+\alpha }\left[\left(x^{\alpha +1}\;+\;{\left(-1\right)}^{\alpha }\right)\ln\left(x\;+\;1\right)\;+\;{\left(-1\right)}^{\alpha }\sum_{n=1}^{\alpha +1}{\left(-1\right)}^{n}\frac{x^{n}}{n}\right], & \mathrm{if}\;\alpha \geq 0,\\ \frac{1}{\ln2}\frac{1}{1+\alpha }\left[\left(x^{\alpha +1}\;+\;{\left(-1\right)}^{\alpha }\right)\ln\left(x+1\right)\;+\;{\left(-1\right)}^{\alpha +1}\ln x\;-\;\sum_{n=\alpha +2}^{-1}{\left(-1\right)}^{n}\frac{x^{n}}{n}\right], & \mathrm{if}\;\alpha <0,\;\alpha \neq -1,\;\;\;\;\;\;\;\\ -{\mathrm{Li}}_{2}\left(-x\right), & \mathrm{if}\;\alpha =-1, \end{array}\right. \end{array}$$

where ${\mathrm{Li}}_{2}\left(\cdot \right)$ is a Spence’s function, we define y(R) as follows,

(A6) $$y\left(R\right)≜E_{2}-E_{1}=\left\{\begin{array}{cc} \frac{1}{\left(\ln K_{2}-\ln K_{1}\right)}\left(\gamma_{1}+\gamma_{2}-K_{1}-K_{2}\right), & \mathrm{if}\;\alpha =-1,\\ \frac{1}{K_{1}^{-1}-K_{2}^{-1}}\left(\ln\gamma_{1}+\ln\gamma_{2}-\ln K_{1}-\ln K_{2}\right), & \mathrm{if}\;\alpha =-2,\\ \frac{\alpha +1}{\left(\alpha +2\right)\left(K_{2}^{\alpha +1}-K_{1}^{\alpha +1}\right)}\left(\gamma_{1}^{\alpha +2}+\gamma_{2}^{\alpha +2}-K_{1}^{\alpha +2}-K_{2}^{\alpha +2}\right), & \mathrm{otherwise}. \end{array}\right.$$

If y(R)≥0, then $E_{2}\geq E_{1}$; thus, policy II is optimal. Otherwise, policy I is optimal. Now, we check whether y(R)≥0 or y(R)<0. By partially differentiating both sides of (A3) and (A4) with respect to *R*, we obtain

(A7) $$\begin{array}{ccc} \frac{\partial \gamma_{1}}{\partial R} & \;=\; & \frac{1}{K\log_{2}\left(1+\gamma_{1}\right)\gamma_{1}^{\alpha }}>0, \end{array}$$

(A8) $$\begin{array}{ccc} \frac{\partial \gamma_{2}}{\partial R} & \;=\; & \frac{-1}{K\log_{2}\left(1+\gamma_{2}\right)\gamma_{2}^{\alpha }}<0. \end{array}$$

Again, by partially differentiating both sides of (A7) and (A8), we obtain the second derivatives of $\gamma_{1}$ and $\gamma_{2}$ with respect to *R* as

(A9) $$\begin{array}{ccc} \frac{\partial^{2}\gamma_{1}}{\partial^{2}R} & \;=\; & -\frac{1}{K}\frac{\partial \gamma_{1}}{\partial R}\frac{1}{\gamma_{1}^{\alpha +1}\log_{2}\left(1+\gamma_{1}\right)}\left\{\frac{\gamma_{1}}{1+\gamma_{1}}\frac{1}{\ln(1+\gamma_{1})}+\alpha \right\}, \end{array}$$

(A10) $$\begin{array}{ccc} \frac{\partial^{2}\gamma_{2}}{\partial^{2}R} & \;=\; & \frac{1}{K}\frac{\partial \gamma_{2}}{\partial R}\frac{1}{\gamma_{2}^{\alpha +1}\log_{2}\left(1+\gamma_{2}\right)}\left\{\frac{\gamma_{2}}{1+\gamma_{2}}\frac{1}{\ln(1+\gamma_{2})}+\alpha \right\}. \end{array}$$

Because $0\leq \frac{z}{1+z}\frac{1}{\ln(1+z)}\leq 1$, we obtain the following new analytical findings from (A9) and (A10).
*If α≥0, $\gamma_{1}^{\prime \prime }\leq 0$, and $\gamma_{2}^{\prime \prime }\leq 0$, then $\gamma_{1}$ and $\gamma_{2}$ are concavely increasing and decreasing over R, respectively. However, if α≤−1, $\gamma_{1}^{\prime \prime }\geq 0$ and $\gamma_{2}^{\prime \prime }\geq 0$, then $\gamma_{1}$ and $\gamma_{2}$ are convexly increasing and decreasing over R, respectively.*
Next, we consider four cases to complete the proof using the above statement.

(i) *Case 1 (α≥0)*: Define $h\left(x\right)≜\frac{K}{\alpha +2}x^{\alpha +2}$. Because $h^{\prime }\left(x\right)=Kx^{\alpha +1}>0$ and $h^{\prime \prime }\left(x\right)=K\left(\alpha +1\right)x^{\alpha }>0$, h(x) is a convex increasing function. From the CFC rule, it is evident that $h(-\gamma_{1})$ and $h(-\gamma_{2})$ are convex functions because $-\gamma_{1}$ and $-\gamma_{2}$ are convex functions owing to the non-negative α. Because $h\left(-\gamma_{1}\right)=\frac{K}{\alpha +2}{\left(-\gamma_{1}\right)}^{\alpha +2}=\frac{K}{\alpha +2}{\left(-1\right)}^{\alpha }{\left(\gamma_{1}\right)}^{\alpha +2}$ is convex, $\frac{K}{\alpha +2}{\left(\gamma_{1}\right)}^{\alpha +2}$ is convex if α is an even integer. In contrast, because $x^{\beta -1}$ is a convex function if $x^{\beta }$ is convex with a positive integer, i.e., β≥2 and x≥0, $\frac{K}{\alpha +2-1}{\left(\gamma_{1}\right)}^{\alpha +2-1}$ is also convex. Therefore, in general, $\frac{K}{\alpha +2}{\left(\gamma_{1}\right)}^{\alpha +2}$ is a convex function for all α≥0. Similarly, we can show that $\frac{K}{\alpha +2}{\left(\gamma_{2}\right)}^{\alpha +2}$ is convex. Consequently, y(R) in (A6) is convex because it denotes the sum of two convex functions if α≥0. Furthermore, because $y\left(0\right)=y\left(R_{\max}\right)=0$, we can show that y(R)≤0 if α≥0, which implies that Policy I is optimal if α≥0. Here, $R_{\max}$ is the maximum achievable rate derived as
  $$R_{\max}=\int_{K_{1}}^{K_{2}}\log_{2}\left(1+g\right)Kf\left(g\right)dg.$$
(ii) *Case 2 (α≤−3)*: Because $h^{\prime }\left(x\right)>0$ and $h^{\prime \prime }\left(x\right)<0$, h(x) is a concave increasing function. Thus, −h(x) is a convex decreasing function. From the CFC rule, $-h(-\gamma_{1})$ and $-h(-\gamma_{2})$ are convex as $-\gamma_{1}$ and $-\gamma_{2}$ are concave functions owing to the negative α. Because $-h\left(-\gamma_{1}\right)=-\frac{K}{\alpha +2}{\left(-\gamma_{1}\right)}^{\alpha +2}=\frac{K}{\alpha +2}{\left(-1\right)}^{\alpha +3}{\left(\gamma_{1}\right)}^{\alpha +2}$ is convex, $\frac{K}{\alpha +2}{\left(\gamma_{1}\right)}^{\alpha +2}$ is convex if α is an odd number. If $x^{\beta }$ is convex when β≤−3 and x≥0, then $x^{\beta -1}$ is convex. Therefore, $\frac{K}{\alpha +2-1}{\left(\gamma_{1}\right)}^{\alpha +2-1}$ is also convex. Therefore, $\frac{K}{\alpha +2}{\left(\gamma_{1}\right)}^{\alpha +2}$ is convex for all α≤−3. Similarly, we can show that $\frac{K}{\alpha +2}{\left(\gamma_{2}\right)}^{\alpha +2}$ is convex for all α≤−3. Consequently, y(R) is convex for all α≤−3 as it denotes the sum of two convex functions. Furthermore, because $y\left(K_{1}\right)=y\left(K_{2}\right)=0$, we can show that y(R)≤0 if α≤−3, which implies that Policy I is optimal if α≤−3.
(iii) *Case 3 (α=−1)*: y(R) is convex because it denotes the sum of two convex functions: $\gamma_{1}$ and $\gamma_{2}$. Note that $\gamma_{1}$ and $\gamma_{2}$ are convex owing to the negative α. Furthermore, because $y\left(0\right)=y\left(R_{\max}\right)=0$, we can show that y(R)≤0 if α=−1, which implies that Policy I is optimal if α=−1.
(iv) *Case 4 (α=−2)*: From (A5), we obtain the following equalities when α=−2:
  (A11) $$\begin{array}{cc} f^{-1}\left(R\right) & =c_{1}\left(-\left(\frac{1}{\gamma_{1}}+1\right)\ln\left(\gamma_{1}+1\right)+\ln\gamma_{1}+c_{2}\right),\\ g^{-1}\left(R\right) & =c_{3}\left(\left(\frac{1}{\gamma_{2}}+1\right)\ln\left(\gamma_{2}+1\right)-\ln\gamma_{2}+c_{4}\right), \end{array}$$
  where $\left\{c_{1}\ldots ,c_{4}\right\}$ are constant values. Because $f^{-1}\left(R\right)$ is a logarithmically increasing concave function, $\gamma_{1}$ is a logarithmically convex function, i.e., $\ln(\gamma_{1})$ is convex. In contrast, $g^{-1}\left(R\right)$ is an exponentially decreasing convex function; thus, $\gamma_{2}$ is a decreasing logarithmically convex function, i.e., $\ln(\gamma_{2})$ is convex. Therefore, y(R) in (A6) is convex. Considering that $y\left(0\right)=y\left(R_{\max}\right)=0$, we can show that y(R)≤0 if α=−2, which implies that policy I is optimal if α=−2.

This completes the proof of this theorem. □
